# Power ultrasound in the meat industry (freezing, cooking and fermentation): Mechanisms, advances and challenges

**DOI:** 10.1016/j.ultsonch.2022.106027

**Published:** 2022-05-06

**Authors:** Mahmoud Soltani Firouz, Hamed Sardari, Peyman Alikhani Chamgordani, Maryam Behjati

**Affiliations:** Department of Agricultural Machinery Engineering, Faculty of Agricultural Engineering and Technology, University of Tehran, Karaj, Iran

**Keywords:** Ultrasound, Meat, Processing, Cavitation, Quality, Promotion

## Abstract

High intensity ultrasound (HIUS) has a wide range of applications in different sectors of food processing. It is a promising and emerging technology demonstrating the potential to promote food processes without or at least damage to the quality of products. Among the processes of the meat industry, freezing, thawing, cooking and fermentation are very sensitive and important, because they have significant effects on product quality and are also very energy and time consuming. This review paper provides an interpretation of high intensity ultrasound (HIUS) applications, a summary of recent outstanding published research and an overview of the freezing/thawing, cooking/frying and fermentation processes in meat and its products assisted by HIUS. The effects, benefits and drawbacks as well as the challenges ahead in the commercialization of this technology in the meat industry are studied. The research results confirmed that the use of HIUS in the meat freezing/thawing, cooking/frying and fermentation in combination with the corresponding processing methods demonstrates a great potential to promote the process, improve the general quality of the final product and reduce the time and energy required. However, many issues remain that require further research to address these challenges. These challenges and subsequent research that is useful for developing and increasing the efficiency of this technology have been reviewed. After the literature review, it is concluded that HIUS may be a useful technology for meat processing because of its significant effects on the quality factors and related process variables that leads to the preservation of the initial nutritional and sensory properties of meat and its products. Of course, research must be continued to eliminate the disadvantages or minimize the undesirable effects of this technology on the final product and to remove barriers to commercialization and optimization of this method.

## Highlights

• Recent advances of high intensity ultrasound (HIUS) are reviewed in meat processing.
• HIUS has significant capabilities in different applications of meat processing.
• HIUS helps to accelerate the process and usually (but not always) improves the quality.
• The efficiency of HIUS depends on power, frequency, temperature and probe properties.
• Further research is necessary to improve HUIS applications in the meat industry.

## Graphical abstract

**Figure:**
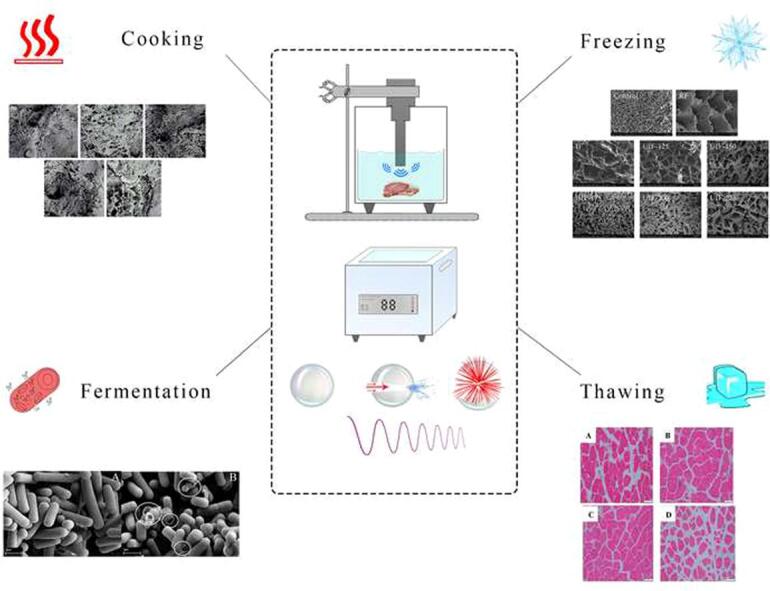

## 1 Introduction

Ultrasound is an innovative technology and it is based on the application of mechanical waves at a frequency ranging from 20 kHz to 10 MHz [1] which can be classified into two frequency ranges: low-intensity ultrasound (LIUS) which uses high frequencies between 100 kHz and 10 MHz at intensities lower than 1 Wcm^−2^, while high-intensity ultrasound (HIUS), uses intensities higher than 1 Wcm^−2^ at low frequencies between 20 kHz and 100 kHz [1].

HIUS has the ability to change the chemical and physical properties of food in filtration, drying, sterilization, extraction, food preservation, emulsification, tempering, bleaching, etc. [2]. The benefits of HIUS applications in the food industry are increased efficiency, improved mass and heat transfer, reduced processing time, increased physical mixing, lower processing temperature, selective extraction, increased yield, etc. The application of the HIUS technology in food processing has achieved good results. Furthermore, HIUS can be used in the “Green Food Processing” strategy as an efficient tool to ensure high-quality and safe food [3].

Due to the non-invasive and non-destructive nature of LIUS, this technology is appropriate for tracking the food processes, providing an assessment of physicochemical changes of food during processing, as well as for food analysis and detection. Different from LIUS, HIUS has a strong physicochemical effect on foods. In recent years, researchers have focused on studying the effects of HIUS on fresh meat processing including the mass transfer or marinating [4], [5], meat tenderizing [6], [7], freezing and thawing[8], [9], drying [10], [11], etc. In meat processing, HIUS can modify the cell membrane helping in drying, curing, and tenderizing tissues. Tenderness, juiciness, and flavor as well as organoleptic properties are the main quality factors of meat [12]. Many studies have shown that HIUS has a positive effect on the quality of meat [9], [13] However, there are reports of adverse effects of this technology on the organoleptic and physicochemical properties of meat. As an example, lipid oxidation in meat processing generally results in the production of off-flavors and off-odors, leading to adverse sensory properties. It has been proved that HIUS encourages oxidation of both, lipid and protein [14], [15]. In addition to collagen fragmenting, as reported by Kuijpers et al. [16] the 20 kHz ultrasonic probe causes the formation of free radicals, this phenomenon leads to oxidation of proteins in beef and loss of solubility of myofibril components [14]. An issue that needs to be addressed is that the absorbed energy of HIUS may lead to heat generation rising to elevated temperatures and subsequently resulting in thermal damage to meat [17], [18]. The effects of HIUS on water holding capacity and drip loss of meat can be evaluated positively or negatively, the changes depend on fiber type and orientation, biochemical properties as well as power and time of ultrasonication.

In this paper, some of the ultrasound applications in the meat industry will be discussed including freezing/thawing, cooking and fermentation and their effect on the quality of products are evaluated.

## 2 Ultrasound-Assisted immersion freezing (UIF)

Freezing is one of the most traditional meat preservation processes that increases the shelf life of meat by inhibiting microbial growth and reducing chemical and enzymatic reactions [19] to retain their initial nutritional properties and sensory characteristics [20], [21]. In frozen meat products, the physicochemical and biochemical activities are slowed down, which are responsible for the loss of nutrients, adverse color, texture changes, and off-flavors [22]. During freezing, microstructural changes affect the quality, texture, and shelf life of meat depending on the size and shape of the ice crystals. It has been found that the quality of food products including color, nutrients, and texture properties are functions of the freezing rate [23]. In conventional methods, freezing problems occur when the freezing rate is low, in which case the ice crystals are large and have sharp edges, which are usually formed in the intercellular space and cause cell damages, finally, this leads to a degradation of the product quality. Also, temperature fluctuations during storage and storage time cause irregular crystals to form, resulting in a decrease in meat quality. Slow freezing and the formation of large ice crystals can cause serious damages to the muscle tissue of the meat, which adversely affects the quality of the meat by losing water in the meat during thawing [24], [25]. Freezing damage to meat is a critical issue, comprising disruption tissue structure, changes in osmotic pressure and functional properties, and unfolding of protein structure [26]. Hence, many researchers focused on this issue to develop novel technologies to control crystallization and to promote freezing efficiency to improve the quality of frozen meats. Ultrasound shows good potential to be applied as a supplementary technology for performance improvement of both freezing and thawing of meat products [22]. Freezing involves lowering the temperature until at least a minimal portion of the water has crystallized into ice. When the freezing rate is high, since the heat transfer rate is high and there is not enough time for water to escape from the meat tissue, crystals are produced in smaller, more uniform sizes and greater numbers inside and outside the cell. Therefore, it prevents the reduction of product quality [24], [25]. One of the methods that can be very helpful in reducing the size of ice crystals and increasing nucleation is HIUS [27]. HIUS promotes the crystallization process by nucleation and breaking ice crystals. HIUS can produce a large number of cavitation bubbles acting as nuclei to form ice crystals. Thus, UIF helps maintaining the quality of frozen meat by increasing the freezing rate and promoting the formation of small and even ice crystals [27]. HIUS can also effectively increase the speed of convective heat transfer and the rapid movement of cavity bubbles also increases heat and mass transfer [27]. Power ultrasound has been shown to be effective in the primary nucleation process as well as in the subsequent crystallization process during freezing, In addition to the ultrasound bubbles produced by ultrasound acting as nuclei for the formation of ice crystals, the strong forces caused by the collapse of the cavity bubbles break the ice crystals into smaller sizes, These crushed crystals then act as new nuclei [28], which lead to an exponential increase in the solidification rate, Therefore, by producing small ice crystals, the quality of the product would be maintained due to the protection of the tissue against damage. The phase change of water molecules is a critical event that leads to the release of latent heat and subsequent crystallization of molecules. Applying HIUS at this time can highly improve nucleation rate and crystal growth leading to the production of more uniformly distributed and smaller ice crystals which are two significant factors affecting the quality of frozen meat tissues. Thus, the UIF treatment has a positive effect on meat quality during freezing [29].

Three functional mechanisms are assumed for ultrasound in accelerating the freezing process: (a) microstreaming generated by the stable acoustic cavitation bubbles without the implosion phenomenon makes intensive collision between micro-particles leading to thinner solid-liquid interface [22], [30]; (b) the instantaneous high pressure in the range of 100 MPa, generated by the implosion of cavitation bubbles leads to high supercooling and the subsequent instantaneous nucleation [31]; (c) the secondary nucleation is formed by small pieces of large dendritic ice crystals shattered by microbubbles [22].

**Fig. 1:**
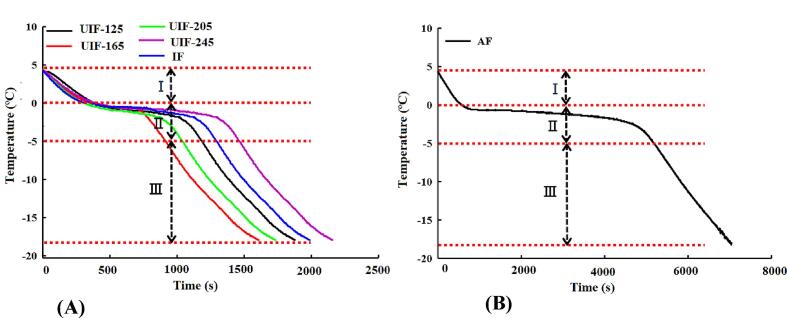
Freezing curves of the chicken breast during different freezing treatments. Air freezing (AF); immersion freezing (IF),; and ultrasound-assisted immersion freezing (UIF), at different ultrasound power levels (125, 165, 205, and 245 W) [34].

As shown in Fig. 1, the freezing process consists of three main parts: the pre-cooling stage (I) which is in the temperature range of 4 °C and −1°C, the phase transition stage (II) which contains the largest ice crystal formation in the range of −1 °C and −5 °C and the last part is sub-cooling stage (III) with the temperature range of −5 °C and −18 °C [32]. Fig. 1A shows the UIF and immersion freezing (IF) curves of the chicken breast during different freezing treatments and Fig. 1B indicates the curve of air freezing (AF), the transition phase is the most important step for evaluating the quality of frozen foods because most of the ice crystals perform in this phase. The shortest phase transition time belongs to 165 W UIF (UIF-165) due to the movements of cavitation bubbles which caused microstreaming leading to intense turbulence flows which help the heat and mass transfer to be improved [33], [34].

At the optimum power of HIUS, the numerous cavitation bubbles act as nuclei promoting the formation of ice crystals and thereby shortening the freezing time as well as minimalizing the phase transition time [34]. The phase transition time of the optimum HIUS assisted freezing is 6 to 8 times lower than that of the air freezing method. This is due to the microstreaming which generates intense turbulence flows promoting the heat and mass transfer during freezing. But ultrasonic powers greater than the optimum value prolong the phase transition time due to the surplus heat generation and decreasing the heat transfer rate [34]. Recent review research confirmed that HIUS greatly increase the probability of nucleation which is critically dependent to the ultrasound intensity. The probability of phase change is closely related to the number of bubble nuclei induced by ultrasound vibration. The size distribution of ice crystals is affected by the temperature of HIUS induced nucleation. Decrease of the nucleation temperature results into smaller ice crystals. Increase in supercooling and HIUS power decrease ice crystals' mean size and increases their mean circularity [33]. As can be seen in Fig. 1 applying HIUS during phase transition stage of freezing promotes the freezing rate compared to the conventional method except for UIF-245, proving that power of HIUS is an important factor in improving heat transfer in phase II. In phase III, HIUS has almost no effect on the rate of temperature decrease and the freezing lines move in parallel. The same results have been reported by Zhang et al. [13] for porcine longissimus muscles freezing. The application of HIUS had no significant effect on the pre-cooling stage (stage I). while, the phase transition time (stage II) increased at first and later decreased with increase in the ultrasonic power. The optimum ultrasound power was 180 W which is due to the promotion of the convective heat transfer rate resulting from both microstreaming and cavitation.

The application of higher intensities than to the optimal value leads to the generation of heat and prolongs the phase transition stage (stage II). Another reason could be the decrease in the contact surface at the coolant/meat sample interface, which is caused by excessive cavitation bubbles. The time of the sub-cooling stage (stage III) is not affected by applying HIUS. Since most of water inside the meat is frozen leading to increasing the thermal conductivity and a rapid temperature reduction. Most uniform distribution of ice crystals and the least structural damage of meat are obtained at the optimal HIUS power [13].

### 2.1 Ultrasound-assisted freezing equipment

**Fig. 2:**
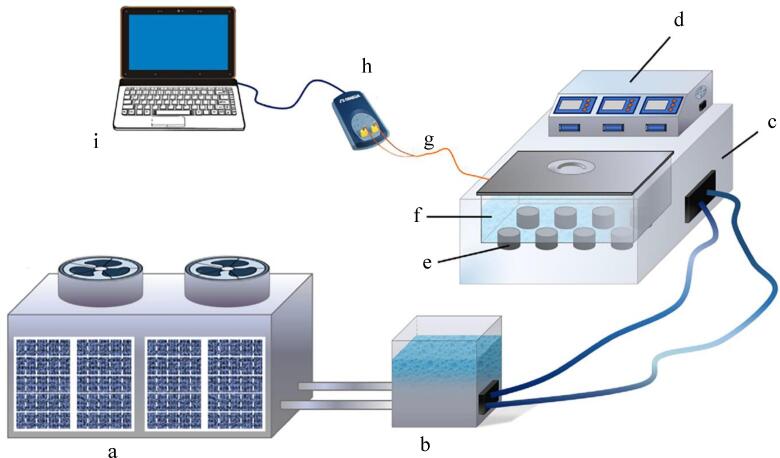
Schematic diagram of the ultrasound-assisted freezing system. a: Refrigeration machine; b: Coolant tank; c: Ultrasound-assisted freezing device; d: Control panel; e: Ultrasound transducer; f: Freezing chamber; g: T-type thermocouple; h: Thermocouple data logger; i: Computer [35].

**Fig. 3:**
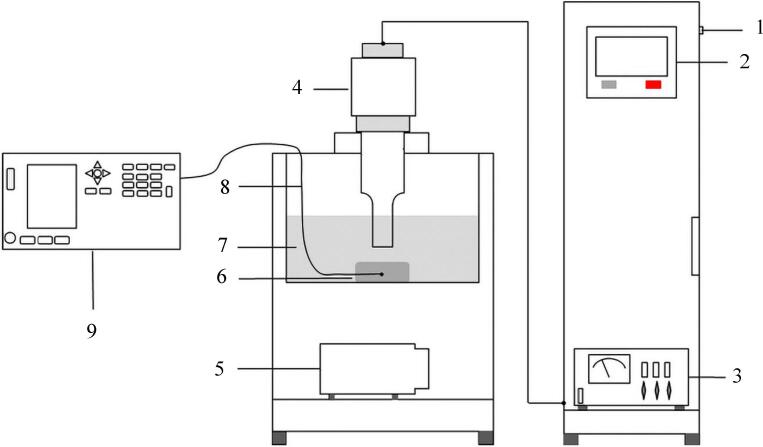
Ultrasound-assisted freezing system (1. Power supply, 2. Ultrasound parameter control panel, 3. Ultrasound generator, 4. Ultrasound probe, 5. Refrigeration compressor, 6. sample, 7. Coolant storage tank, 8. K-type thermocouple, 9. Temperature testing instrument) [36].

Two main ultrasound systems have been used for meat processing: Ultrasonic bath and ultrasonic probe. Ultrasonic bath is the most typical equipment, as shown in Fig. 2, consists of several main parts: the stainless-steel freezing tank, the transducers attached to its base which have the ability to convert electrical energy to ultrasound waves, electrical pulse generator that supplies electrical power to the transducer and the data collection system [35], [36]. In the freezing tank, the coolant and the samples are placed, samples are floating in the liquid medium and the ultrasound wave propagates evenly in the coolant medium. The power generator permits to adjustment of sonication power, duration, duty cycle, etc. Such ultrasonic baths generally work at a frequency in the range of 40 kHz, In addition, the dissipation of the ultrasound power by the medium contained in the bath is a weakness of ultrasound baths [35]. The ultrasonic probe as shown in Fig. 3 includes a booster horn that increases the sonication amplitude and transducers that bonded to the probe. The acoustic waves can directly transfer using ultrasonic horn into the liquid medium, the duty of the sonotrode is to radiate the waves into the medium. By comparing of the bath and probe systems, it should be noted the power intensity of the probe is approximately 100 times higher than the bath system [37].

**Fig. 4:**
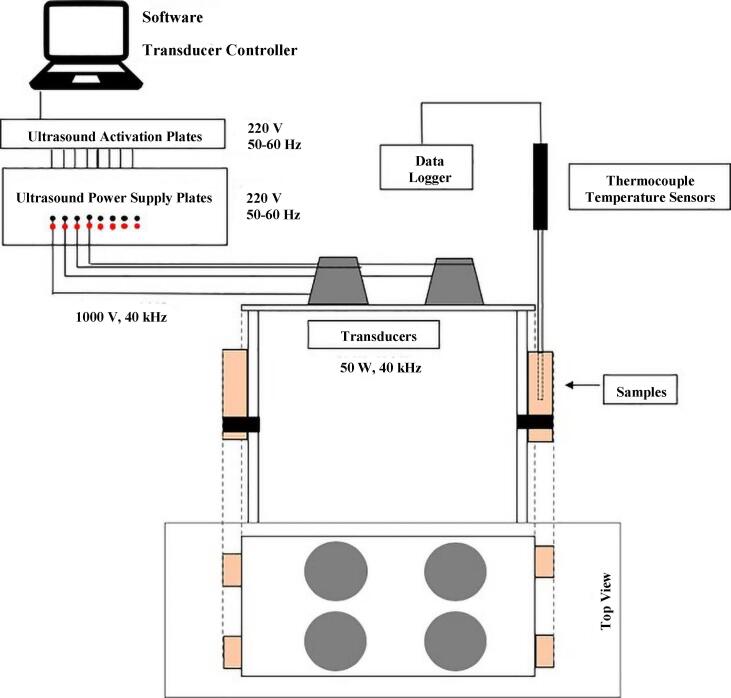
The direct ultrasound-assisted freezing system developed by [8] for chicken breast freezing.

Most studies have focused on the UIF systems using ultrasonic baths, the product is immersed in a liquid medium which is usually ethanol, ethylene glycol, and glycerol [9], [34]. In such immersion systems, HIUS propagates through the liquid medium which generates cavitation, micro jets, and streaming improving heat and mass transfer. The main issue in applying these systems is chemical effects and producing free radicals. A strategy that can overcome this issue is direct contact ultrasound-assisted freezing. This method is based on the presence of an ultrasonic transducer or a horn connected to the plate on which the samples are placed so that the generated vibration directly transmits to the samples [8]. This system is interesting for meat freezing since the liquid coolant is eliminated and cold air is applied instead, which increases the ability of such systems to be industrialized and facilitates the application of this technology is currently static freezing lines used in the industry. For meat, the direct contact ultrasound-assisted freezing method minimizes the chemical effects as well as lipid oxidation since cavitation is very unlikely to occur [8]. Fig. 4 show the direct UIF system developed by [8]. They found this method did not affect in vitro protein digestibility of chicken meat. Nevertheless, further studies are necessary to improve the performance of such systems.

### 2.2 Results of UIF process

The use of HIUS in the freezing of food, including meat, can reduce the damages to the product that occurs in the traditional methods, and also can increase the quality of the product. Ultrasound has received a lot of attention in recent years, and its use in food freezing has shown promising benefits. Sun et al. [26] studied changes in the emulsifying and gel properties of myofibrillar proteins in common carps (Cyprinus carpio) frozen by UIF. They found that UIF at 175 W of HIUS (UIF-175) effectively prevents the reduction of protein solubility, emulsion activity index, absolute Zeta potential storage modulus, and loss modulus arising from freezing. The treated samples by UIF-175 show a higher gel strength, denser, and more uniform gel network structure than the conventionally treated samples.

The shear forces generated by the cavitation bubbles can also destroy hydrogen bonds as well as electrostatic and hydrophobic interactions which leads to a reduction in the protein particle size [26]. But a suitable ultrasonic power should be applied to be able to enhance the electrostatic repulsion between the particles and to prevent the aggregation of the protein, thereby improving the stability of the protein dispersion. The effect of UIF-180 on the microstructure, quality, and water distribution of porcine longissimus muscles during frozen storage was evaluated by M. Zhang et al. [38]. They found UIF-180 decreased water migration and lipid oxidation during frozen storage as well as higher redness than that of the conventional methods. Also, the UIF-180 treatment was more effective in maintaining the original muscle tissue than the conventional methods.

Reduction of thawing loss of meat with the help of HIUS is related to the formation of small and uniform ice crystals, resulting in less damage to the cell walls. While, in conventional freezing, migration of water to the extracellular space is inevitable, which leads to the formation of irregular and large ice crystals and thereby the destruction of cell walls and weakening the ability to reabsorb molten water into the cells after thawing. Table 1 represents the results with optimal power of the Ultrasound-assisted freezing process for preserving the quality of meats.

**Table 1:** Some applications of ultrasound-assisted freezing in the meat industry.

Product	Method	Optimal conditions	Results	Reference
Chicken breast	Ultrasound-assisted immersion freezing, ultrasonic bath equipment	165 W, 30 kHz, 8 min, with a 30 s on/30 s off cycle	Smaller ice crystals; enhanced the freezing rate, reduced the thawing and cooking losses, decreased the mobility of the immobilized and free water, and maintained the protein thermal stability.	[34]
Rabbit meat	High intensity ultrasound (40 kHz)	–	Not recommended for long exposure time post-freezing due to increasing the weight loss and meat toughening, for short exposure time water holding capacity was significantly increased.	[39]
Common carp (*Cyprinus carpio*)	Ultrasound-assisted immersion freezing, the pilot-scale ultrasonic bath system	175 W, 30 kHz, pulse mode 30 s on/30 s off.	Smaller and more uniform ice crystals, maintain the integrity of the muscle tissue, reducing thawing and cooking loss; excess ultrasonic power results in larger ice crystals.	[9]
Porcine longissimus muscles	Ultrasound-assisted immersion freezing, the pilot-scale ultrasonic bath system with a coolant including 95% ethanol and 5% fluoride	180 W, 30 kHz, a 30 sec on/30 sec off cycle was applied for 8 min when the temperature of the chops decreased to 0 °C. Samples were exposed to HIUS at 30 s intervals to prevent excessive heat generation from continuous HIUS.	Reduced the size of ice crystals and more uniform distribution, reduction in thawing loss, reduce the mobility and loss of immobilized and free water. Higher intensities of HUIS than to the optimum value led to the generation of heat at the surface of the meat limiting the possibility of lower final temperature achieving.	[13]

**Fig. 5:**
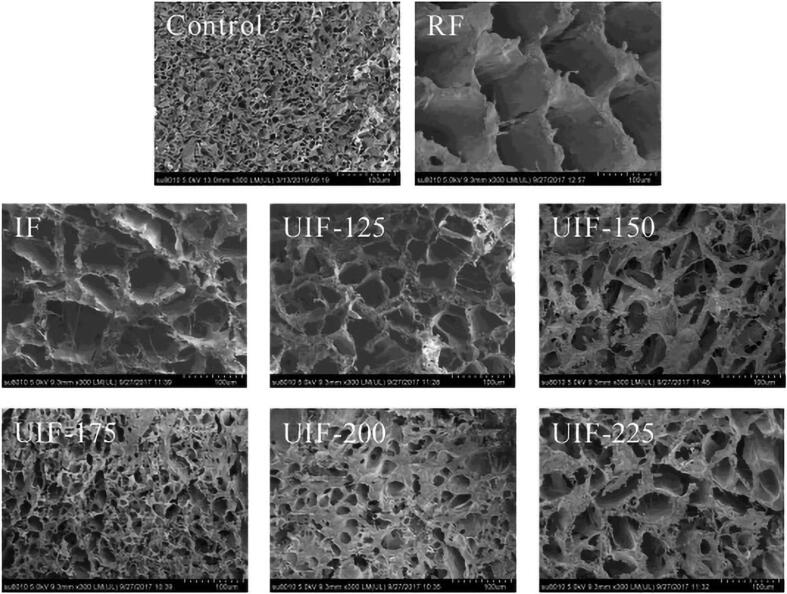
SEM images of carp muscles with different cryopreservation treatments (magnification: 300x). RF: refrigerator freezer; IF: immersion freezing; UIF: ultrasound immersion freezing at various ultrasound powers (125 W, 150 W, 175 W, 200 W, and 225 W) [9].

Another way for evaluating the performance of the UIF systems is to use the scanning electron microscope (SEM) imaging which has a magnification of 10 to 500,000 times with a resolution of less than 1 to 20 μm. SEM is used to analyze the morphology of microstructures and to identify chemical compounds, as shown in Fig. 5 which represents the SEM imaging of carp muscles, the control sample has smaller and denser pores. In the refrigerator freezer (RF), due to the lower speed of freezing and the formation of larger ice crystals inside and outside the cells, they have the largest size in the pores. The pores are also related to the size and distribution of ice crystals which both are controlled by the time or freezing speed. Rapid freezing results in the formation of small and uniform ice crystals, while slow freezing usually produces larger ice crystals, which leads to cell destruction [9].

Compared to the RF samples, the UIF samples had smaller pores, which confirms the formation of smaller ice crystals due to the speed of heat transfer in the liquid. The samples in 175 W UIF (UIF-175) condition, have the smallest and most uniform ice crystals according to the shape and size of the respective pores. It should be noted that too little or too much ultrasonic powers cause large ice crystals to form and damage the microstructure of the meat, Therefore, at 200 W and 225 W, due to excessive power and at 125 W and 150 W due to insufficient power input, the crystals were larger than the optimal sample in 175 W [9]. These results have been obtained at the constant frequency of 30 kHz, the ultrasonic intermittent mode of 30 s on/30 s off cycle which once the sample center temperature decreased to 0 °C, HIUS irradiation was started and lasted for 9 min. However, effect of ultrasonic power was studied, but effect of the time of exposure and the frequency of HIUS have not been investigated. The time of ultrasound exposure is usually a function of the completion of the freezing process, in which the HIUS- assisted freezing process is continued until the geometric center of the sample reaches to −18° C [13], [40], or in some research the time of exposure is considered constant [34], [41]. The same experimental conditions and freezing system has been applied for chicken breast freezing [34], but with the difference that the ultrasonic powers used were 125, 165, 205, 245 W. Zhang et al. [34] also reported the similar results. With increasing the power of HIUS, the total freezing time of the UIF samples first decreased and then increased, while the total freezing time at the UIF-165 treatment was the lowest as 38.2% lower than that of the IF treatment which was due to the movement of the cavitation bubbles produced by UF-165 arising from microstreaming and further intense turbulence flows promoting the heat transfer during freezing. They also reported excessive ultrasonic power increased the phase transition time due to the formation of excessive cavitation bubbles and heat generation, resulting in a decrease in the heat transfer and freezing rate. Microstructure analysis of the chicken breast samples confirmed that for the AF samples, the holes left by the ice crystal were large and irregular which was due to the slow freezing rate and formation of large and irregular ice crystals. while the cavities left by the ice crystals in the microstructure of UIF-165 samples were the smallest and had the most uniform distribution. For excessive ultrasonic power levels (205 and 245 W), an increase in the size of pores was reported.

Due to the structural, physicochemical and physicomechanical differences in meat products, the frequency, power and intensity of HIUS suitable for each product may be different, therefore, to select the optimum ultrasonic parameters, individual experiments must be performed to optimize the HUIS-assisted freezing process [22]. But most authors applied single-frequency ultrasound in meat and meat products freezing. In such studies, ultrasound power and intensity were often considered as variables and the ultrasound frequency has been considered as a constant parameter. Recently, multi-frequency ultrasonic system has been applied for cultured large yellow croaker freezing [40]. The size of the ice crystals is an important parameter affecting the microstructure and thawing process of frozen fish. The traditional freezing methods result in the formation of large ice crystals and subsequently destruction to the fish tissue. Therefore, accelerating the freezing rates and regulating the ice nucleation processes would be beneficial to better maintain the quality of frozen yellow croakers. Ma et al. [40] studied the effects of multi-frequency HIUS on the freezing rates, quality attributes, and structure of cultured large yellow croaker, owing to that the multi-frequency ultrasonication treatment presents better results than mono-frequency ultrasonic treatment on the freezing rate, quality properties, and microstructure of meat. They found the 175 W triple UIF at 20, 28 and 40 kHz was more effective than that of single UIF at 20 kHz and dual UIF at 20 and 28 kHz.

Ma et al. [40] proposed four aspects for the mechanism of the HIUS treatment in accelerating the freezing process: (i) the cavitation effect, the cavitation bubbles implode during the HIUS propagation leading to the instantaneously generated local high pressure which can increase the supercooling degree, providing a great driving force for nucleation, (ii) the generated microstreaming results in strong turbulence helping to induce nucleation and promotes heat and mass transfer efficiency, (iii) cavitation bubbles can appear as ice nuclei to promote primary nucleation, (iv) the dendritic ice crystals are shattered into smaller fragments due to the effect of microstreaming, thereby improving secondary nucleation.

In the conventional freezing methods, the formation of large ice crystals exacerbates mechanical damages to the cells, as well as protein denaturation and thawing losses. After freezing and during storage, the size of the crystals continues to increase, which increases the quality losses. Sun et al. [41] found that the degree of ice crystal growth during storage in the UIF method was less than that of the conventional ones which prove that UIF can reduce the increased rate of ice crystal formation during frozen storage.

### 2.3 UIF in brief

In summary, as described above, it can be defined as follows: quick freezing is generally more desirable because of less damage to the product cells and textures (especially protein tissue) and retaining the initial nutritional properties of a product [21] due to lower size of ice crystals that are uniformly distributed across the tissue, but on the other side and with slow freezing, larger ice crystals are placed in extracellular regions [42], [43], [44], [45], [46] which cause the damages and quality loss of the product, Thus, the quality of slow-frozen food is rather poor. Therefore, it is desirable to develop technologies that help to increase the rate of freezing.

UIF can improve and enhance the freezing rate, can reduce the size of the ice crystals, and can control the distribution of the crystals [47], leading to the improvement of the quality of the frozen products [48]. The food freezing process depends on several factors such as the rate of freezing and the state of water in a product which are the most important and influential factors on the size and distribution of ice crystals in the product. Although recent research shows a good and positive effect of the use of ultrasound technology on the quality and preservation of food nutrients, UIF still needs further researches. Furthermore, it should be noted that the UIF method has some disadvantages, for example HIUS may damages or alters the structure of some compounds in meats during the freezing process due to the formation of •H and •OH free radicals [9], [38]. The liquid medium that is used as a coolant in the ultrasonic systems dissipates the ultrasonic power which may cause insufficient and slow freezing.

It is worth mentioning that the formation of the small ice crystals and uniform distribution as well as improvement of freezing by HIUS can reduce cooking loss, color changes and texture damage [22], [40] and organoleptic properties [13] of meat products.

## 3 Ultrasound-assisted thawing (UTH)

Frozen meat products usually need to be thawed for consumption and further processing. The conventional thawing methods are usually based on hot air blast, hot water, and vacuum. The conventional thawing process affects the quality of frozen meat. The main drawbacks of these methods are low efficiency, high energy and time consumption, and quality losses. An inefficient thawing process usually deteriorates the quality of the meat and highly accelerates microbial growth and activities. The conventional thawing methods have also some drawbacks including dripping and surface heating as well as the severe denaturation of protein. In meat thawing, the damaged cell membranes leak out intracellular fluid and on the other hand, the water held by capillary force decreases due to the destruction of muscle fibers. In addition, fat oxidation and subsequently protein degeneration, resulting in a reduced water-binding ability of protein. The cooking loss had a similar change trend to thawing loss [41]. Today, researchers focus on supplementary processes to improve the meat thawing treatment. HIUS as an assistant technology has attracted the attention of scientists to study on the application of HIUS in thawing for frozen meat to overcome the mentioned disadvantages [49]. Ultrasound-assisted thawing (UTH) as one of the useful applications of ultrasound in the food industry is an innovative technology that helps to accelerate the thawing rate as well as retaining its initial quality properties with the help of the heat generated inside the frozen food materials [50], [51]. HIUS by producing high-speed jets increases the temperature of the thawing water and as well as generating asymmetric bubble collapse subsequently improving heat transfer [22] and shortens the thawing process, accordingly.

A wide range of research has been conducted on investigating the effects of HIUS on the thawing rate, the thawing efficiency as well as the quality characteristics of the meat and its products during the thawing process [52], [53], [54], [55]. Several factors including frequency, sample orientation, intensity, and the composition of the thawed products affect the UTH process. A critical factor that may improve the UTH process is frequency. To prevent the poor ultrasound penetration and achieving acceptable surface temperatures, frequency should be applied in a proper domain. It should be noted that by exceeding the maximum value suitable for the frequency, surface overheating may occur [22]. The most important issue in this regard is the design and manufacturing of multi-frequency sonotrodes. Ultrasonic soundtracks usually operate at a specific frequency called the resonance frequency, which makes it impossible to develop sonotrodes capable of covering a continuous range of frequencies. The conventional ultrasonication system are usually manufactured in the standard frequencies in the ranges of 20–40 kHz which make it difficult to study the effect of ultrasound frequency on the performance of the UTH technique.

The effect of UTH in the range of 25 kHz on the thawing rate and thawing time of pork meat was analyzed [56] which results in reducing by 87% the time required for thawing in comparison with the air thawing, also thawing rate was improved with 0.2, 0.4 and 0.6 W/cm^2^ ultrasound intensity which causes improvements the rates by 0.46 and 0.57 and 0.84 °C/min, respectively. The texture and functional properties of pork meat were remained without impairing at the end of the treatment. In another study conducted by Gambuteanu and Alexe [57], no significant damage to the texture of the pork meat were reported.

**Fig. 6:**
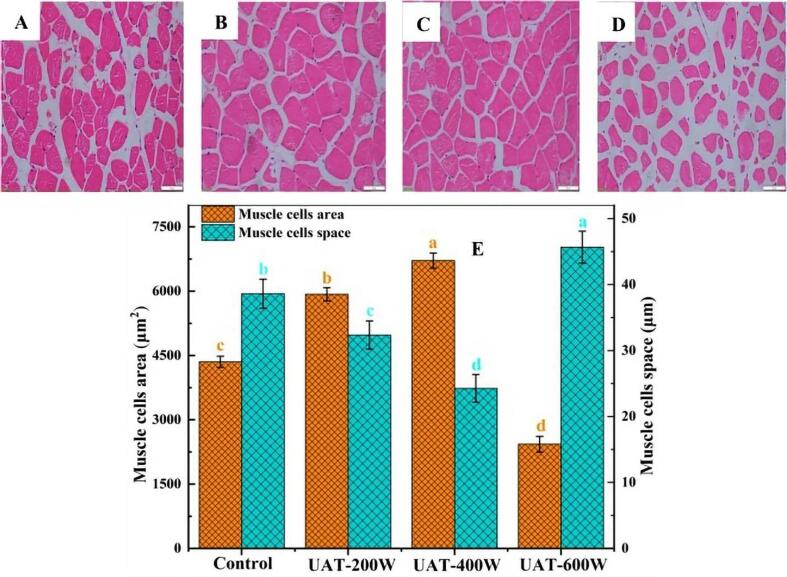
Microstructure of frozen white yak meat with different thawing treatments. UAT, ultrasound-assisted immersion thawing at different ultrasound powers (200, 400 and 600 W) [53].

During the UTH process, the ultrasound cavitation can change the microstructure of meats, as shown in Fig. 6. The study of Guo et al. [53] demonstrates the effect of UTH on the microstructures of white yak meat. The power levels of 0, 200, 400, and 600 W for ultrasonication (frequency of 20 kHz) were used for thawing indicating that in ultrasonication in 200 and 400 W the boundaries get clearer. The treatment with power of 400 W demonstrated the smallest muscle fiber space and the greatest muscle fiber area as well as increasing the thawing efficiency and minimizing the unwanted damages. Furthermore, by applying the power of 600 W, the muscle fiber membranes were ruptured because of the significant value of muscle cell space. This result confirms that optimization of the UTH process of meat thawing seems to be inevitable. Ultrasonication powers greater than that of the optimum value may have a detrimental effect on the texture and structure of the meat. However, consumption of more energy and overheating are also among its effects. Some applications of ultrasound-assisted thawing in the meat industry represented in Table 2.

**Table 2:** Some applications of ultrasound-assisted thawing in the meat industry.

Product	Method	Optimal conditions	Results	Reference
Bighead carp (Aristichthys nobilis) fillets	Ultrasound-assisted water immersion thawing, ultrasonic bath.	28 kHz, intensity of 0.135 W/mL with no pulse-off. For all samples, when the central temperature reached 0 °C, thawing was stopped.	The proposed method dramatically shortens thawing time than the conventional thawing methods; preserves color and pH, and inhibits lipid oxidation; massive water loss and muscle destruction were observed.	[54]
Pork loin	Ultrasound in brine (2% NaCl, w/v)	150 W, 40 kHz. The meat was trimmed of all visible fat and connective tissue and cut into a rectangular form (4 × 2 × 6 cm) parallel to the muscle fiber direction.	Reduced cooking loss and improved tenderness, compared to the control samples	[58]
White yak meat	Ultrasonic bath, power levels of 0, 200, 400, and 600 W (20 kHz)	400 W, with no pulse-off.	Shortening the thawing time of white yak meat, retaining the quality and nutrition of meat	[53]
Common carp (Cyprinus carpio)	Ultrasonic-assisted immersion thawing at 0, 100, 300, and 500 W, ultrasonic bath	300 W, 30 kHz, distilled water was used as the immersion liquid.	The thawing time decreased with the increase of ultrasonic power, However, the thawing and cooking losses at first decreased and then increased. The optimum ultrasonic power was 300 W which was beneficial for quality preservation during thawing	[55]

### 3.1 Challenges

Ultrasound as a useful and innovative technology that changes the physical and chemical structures of the products, helps to improve the quality, however, has challenges that can be mentioned as follows: The heat generated by the thermal effect of ultrasound is absorbed by the liquid medium, which can neutralize some of the positive effects of ultrasound and even negatively affect the freezing process, for example, the rate of freezing; If the ultrasound irradiation is in the continuous mode, it can generate a lot of heat that prevents ice crystals to form; Non-uniform distribution of ultrasonic power in the freezing system causes an unbalanced distribution of HIUS to different parts of the food; Since the long duration of sonication is undesirable for nucleation due to the heat generation, short-time ultrasonication is also leading to insufficient nucleation. Therefore, optimization of the sonication time for different applications is inevitable; Meat products are usually non-homogeneous and extremely attenuating materials, which make it difficult for ultrasound waves to transmit through the material, due to the inability to penetrate the inner parts of the product and absorption of HIUS by the outer layers. Localized heating and overheating is a common phenomenon in ultrasonication; During thawing, due to the overheating arising from HIUS, fast and localized temperature increases maybe happen on the surface of frozen meat, which results in a superficial burn, while the inner part of the product has not yet been thawed; However, some researchers proposed the mechanism of HIUS-assisted freezing, including the mechanical, thermal and cavitation effect and heterogeneous nucleation, but the conducted research studies have not provided an unified understanding of the HIUS-assisted freezing mechanism, and its mechanism is not precisely specified; Standardization of the HIUS-assisted freezing process and product variables is a major challenge to scale this technology for industrialization. Ultrasonic parameters and sample variables in research studies are usually different and are not defined according to a standard protocol. Differences in the frequency, power and intensity of ultrasound, as well as the duration and mode of sonication, in studies lead to different results that make the industrialization of this technology difficult for a specific product. Sample properties (including muscle composition and type of muscle, dimensional characteristics, etc.), and coolant medium and its properties (viscosity, working temperature, etc.) are also needed to be standardized to allow for scaling up this technology to industrial levels.

### 3.2 Future trends

According to the mentioned issues, it is necessary to establish UIF systems with uniform power distribution and also to optimize the process parameters. Further studies and optimization of such systems help the use of this technology on larger scales for commercial applications.

Although significant progress has been made in the field of ultrasound-assisted freezing/thawing, this technology is still in laboratory scales more efforts should be made for scaling up to becomes appropriate for commercialized applications. Therefore, further studies should focus on the mentioned issue to propose the optimized HIUS-assisted freezing/thawing process.

## 4 Ultrasound-assisted cooking

Meat quality is a compound of sensory, physicochemical, toxicological and technological properties of meat. Meat quality is significantly affected by its cooking methods. Sensory properties are key quality factors that drastically change in the cooking process, while they directly affect the market [59]. Thermal processing may result in the quality degradation of meat products (nutrients, flavor, color, etc.). On the other hand, the demand for “chemical-free” meat products is growing. Thus, it is very imperative to develop innovative methods to improve the quality of processed cooked meat products. Hence, there is a growing interest in non-thermal processing methods that have least effects on the nutritional and sensory properties of meat [6]. Flavor is a most influential factor for consumers to qualify a meat product for purchasing. The traditional cooking processes usually consume a lot of energy and may destroy the flavor of the food. While, the flavor quality of meatballs improved a lot by ultrasonic-assisted frying [60].

In cooking process, physicochemical and nutritional modifications take place leading to the formation of characteristic meat aroma and organoleptic properties. A lot of research has been conducted to propose a convenient cooking technology to improve the quality of meat products as well as the efficiency of this processing method [61], [62], [63], [64].

Ultrasound as a burgeoning technology demonstrates a good potential to improve the cooking process of meat. Some researchers proved that HIUS does not have an adverse effect on the color of beef [65], [66], rabbit meat [4] and pork [67]. Ultrasound assisted cooking is one of the applications of ultrasound in the meat industry as well. In the traditional cooking methods at high temperatures, while the exterior part of food is going to get overcooked, the interior part may be not cooked as well. While, HIUS has the ability to provide modified heat transfer rates which is the key factor to solve the cooking challenge. HIUS application as a pre-treatment on meat is a topic of interest in food processing. Also, there is a great interest in applying HIUS as a simultaneous process during cooking.

### 4.1 Ultrasound-assisted cooking equipment

**Fig. 7:**
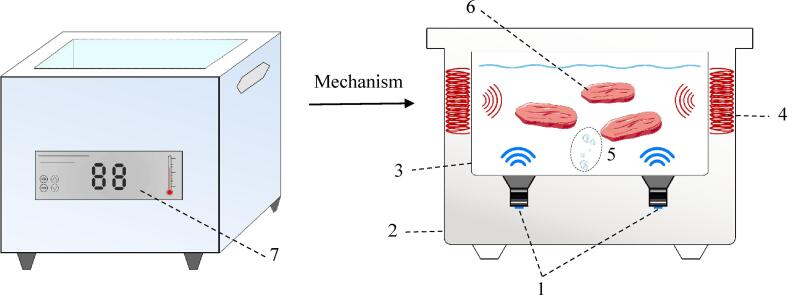
HIUS- assisted cooking bath, (1) Ultrasonic transducers; (2) System housing; 3: Stainless-steel container; (4) Heat generator element; (5) Cavitation phenomenon; (6) Meat sample; (7) Control panel.

Ultrasonic bath and ultrasonic probe are two main systems used in HIUS-assisted meat processing. Fig. 7 shows a HIUS-assisted cooking bath in which the electrical element acts as a heat generator and the HIUS transducers emit ultrasonic wave throughout the liquid medium. During the cooking process, samples immersing in water or brine get sonicated by the transducers placed beneath the tank. The temperature, power, duty cycle, mode of action (continues or pulsed) and the time of sonication are adjustable using the control panel. Cichoski et al [68] used an ultrasonic bath for mortadella cooking at 25 kHz, 50% and 100% amplitude of the ultrasonic wave, corresponding power of 301 W and 462 W, to study on the volatile compounds, oxidative permanence and sensory properties. The treatment with 100% amplitude showed a greater number and diversity of compounds compared to the treatment with 50% amplitude. While 100% amplitude caused a subtractive oxidative stability time, it was higher than the shelf life appointed for the mortadella produced by the industry. The inequality of oxidative stability index between treatments may refer to higher intensity used in the treatment with 100% amplitude which leads to disintegration of fat globules. The increscent number of fat globules has an inverse correlation with the oxidative stability of the product because of greater interaction between unsaturated fatty acids and free radicals [69]. Finally, they could conclude that HIUS-assisted cooking using the ultrasonic bath can lead to a reduction in cooking time with no changes in the sensory properties.

**Fig. 8:**
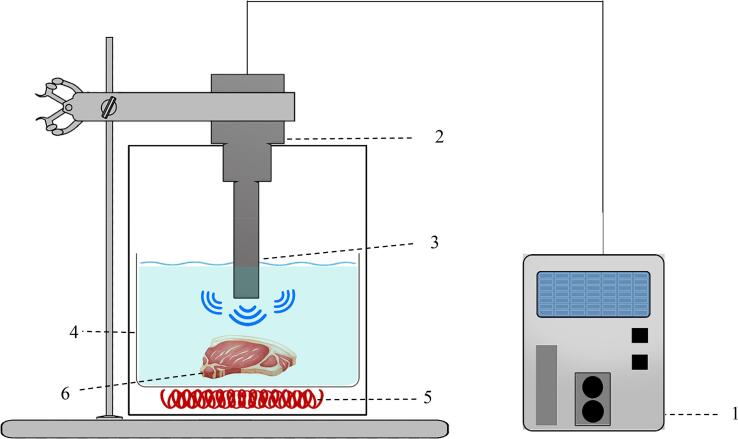
Probe -based HIUS- assisted cooking system. (1) Ultrasound generator and control panel; 2) Ultrasonic probe; (3) Horn; (4) Container; (5) Heat generator; (6) Meat sample.

Fig. 8 shows the ultrasonic probe system applied for meat cooking. The tip of the probe is placed in water or brine inside a container, while the meat samples are immersed in the liquid. The meat samples are usually placed directly into the ultrasound systems without packaging (Fig. 7 and Fig. 8) [60], [66], [70], [71], [72].

The control panel of the system allows to adapt the duration of sonication, the power of sonication, mode of action, etc. In addition, the temperature is controlled by an autonomous heat generator beneath the container. In this case, the samples get sonicated directly by the horn.

### 4.2 Applications of Ultrasound-assisted cooking in meat products

According to [14], HIUS appears to be a forthcoming technology for the meat cooking process, without changing the color of the final product. HIUS leads to the accumulation of collagen in the extracellular space of the matrix of meat, while not causing any change in the color of the meat [73]. The application of HIUS in meat cooking delays the changes of flavor profiles by inhibiting microorganisms during cold storage [66]. It has been proved that HIUS-assisted cooking of meat is faster and more efficient in terms of energy consumption than that of conventional methods and can improve the texture of final product in comparison with the convection cooking method. HIUS-assisted cooking decreases two to five times cooking losses than that meat cooked by conventional methods.

The conventional cooking of spiced beef usually takes a long time for stewed soup to adequately penetrate to beef. The extended cooking time needs more energy consumption which leads to an increase in costs. Zhang et al. [66] applied HIUS in the cooking process of spiced beef to prolong shelf life of the product and studied its effects on the quality properties during cold storage. They reported that in control samples, the total viable count (TVC) increased by 3.77 log10 (cfu/g) after 28 days of storage, while in ultrasonic groups (400, 600, or 800 W), the TVCs were all less than 0 log10 (cfu/g) throughout storage period. This confirms that the HIUS treatment effectively inhibited microorganisms leading to the extension of shelf life of cooked spiced beef. Also, a decrease in the degree of lipid oxidation was observed during cold storage preventing the formation of off- flavor in spiced cooked beef. Ultrasound-assisted cooking decreased protein degradation during the cold storage of spiced beef. Hardness and chewiness of the HIUS-treated beef was lower than that of control samples and significantly decreased as the ultrasonic power was increased. This is due to the destroying the integrity of muscle and the tenderization effect of HIUS. Nevertheless, the decline of hardness was delayed in the HIUS-treated beef compared with control which confirms the preservative effect of HIUS on the stored HIUS-treated cooked beef. It should be noted that higher HIUS powers produce more heat and nonenzymatic browning (Maillard and caramelization reactions) leading to the decrease of L* value.

The cooking process of emulsified meat products is usually a time-consuming as well as a critical process that determines shelf life and quality of such products. The cooking process promotes functional and structural modifications in the myofibrillar proteins [74]. The application of HIUS in the cooking of emulsified meat products has demonstrated excellent capabilities. da Silva et al. [74] used an ultrasonic bath at 25 kHz, sweep mode and 100% amplitude of operation to reduce the cooking time of mortadellas during the production process. The mortadella samples were cooked using the ultrasonic bath and hot water at 60, 65 and 76 °C. The HIUS-assisted cooking, was performed with a 50% shorter cooking time than to the conventional method for each temperature. Compared to the conventional process, the HIUS-assisted cooking reduced the time required for the internal temperature of mortadellas to reach 73 °C by 30%, which was due to the cavitation phenomenon subsequently improving heat transfer. Also, HIUS resulted in a reduction of about 50% of the total cooking time. The HIUS-assisted cooking not only did not increase the protein oxidation of mortadellas, but also provided lower protein oxidation than the conventional method. An issue that reported by da Silva et al. [74] is that the HIUS-assisted cooking provides higher hardness values as well as chewiness, springiness and cohesiveness, compared to the conventional method. This is due to the cavitation phenomenon which leads to modifications in the conformation and unfolding of proteins [75] and subsequently affecting the formation of the more resistant three-dimensional protein network [17] which led to the promotion of mechanical properties. Although HIUS did not affect the color and the microbiological quality of mortadellas, but further studies would be helpful to study the impact of this method on the sensory properties and customer friendliness of this product.

HIUS by producing the hydroxyl radicals and hydrogen peroxide with strong oxidizing abilities may lead to the lipid and protein oxidation of meat [76] which are the most effective degradations related to the flavor and aroma of meat. Zou et al. [70] aimed to study the capabilities of HIUS assisted cooking on the flavor, taste and chemical profiles of spiced beef. Beef blocks were simultaneously immersed in boiling water and exposed to 20 kHz HIUS with the power of 0, 400, 600, 800 and 1000 W. The cooking process was set on 120 min. By increasing the ultrasonic power, the absorption rate of sodium chloride (NaCl) was increased and eventually stabilized during cooking. While, the conventional cooking of spiced beef needs to be heated much longer to achieve the same NaCl content. This is due to that the HIUS treatment can increase the permeability of meat tissue by creating micro-channels in the tissue which results to the promotion of the diffusion coefficients of NaCl and thereby an improved saline taste. The same effect was observed for sugar content of beef blocks. It is worth noting that the excessive heat generated by the application of HIUS more than the optimal level causes the sugar to participate more in the Millard reaction and caramelization reaction eventually producing some aromatic materials.

During the cooking process, the lipid melts and outflows from the meat and releases some lipid-related volatile compounds which can produce the aroma to the cooked meat products [77]. Cichoski et al. [68] studied the effect of HIUS-assisted cooking on the oxidative stability, the volatile compounds and the sensory properties of mortadella. They found that the presence of the volatile compounds from lipid autoxidation in the ultrasound-treated samples may be due to the cavitation phenomenon which produces •H and •OH by breaking up the water molecules that act as initiators of lipid oxidation and produce hydroperoxides (ROOH). The different volatile compounds from spices identified in the ultrasound-treated samples, were not detected in the control ones, which is due to the generating microchannel and micro-agitation on the surface and the interior the samples thereby the enhanced extraction and distribution of the volatile compounds from spices used in the mortadella.

It has been proved that HIUS can promote the permeability of meat to the salt and other substances in the soup [78] due to the mechanical and cavitation effects which may destruct the beef. The myofibre matrix of meat is complex which slows down the penetration of sodium chloride into meat. The mechanical effects of HIUS can destroy the structural integrity of myofibrils allowing to accommodate more water, which increases the water retention of meat. The myofibril framework destruction increases with the HIUS power due to the ultrasonic mechanical effects [76]. In HIUS-assisted cooking of meat, the increased water holding capacity (WHC) is due to the large gap between the myofibrils produced by HIUS. However, the function of sodium chloride in brine should not be ignored which is responsible of the myofibrillar proteins swelling so that more protein side chains combine with water [71].

As a method of cooking, HIUS-assisted frying was recently found to be effective to improve the total flavor of meat. Zhang et al. [60] applied HIUS for 12 min at 160 °C with different powers (0, 200, 400, 600 and 800 W) on meatballs. The results have been analyzed using an electrical nose and interpreted by employing Principal Component Analysis (PCA). The non-sonicated group was obviously different from the sonicated groups proving that ultrasound has a significant effect on the total flavor of the meatballs. Also, among the sonicated groups, the treatment of 200 W distantly located from the other groups portending different flavor properties.

HIUS-assisted frying has also been proved to enhance the overall quality of meatballs as described by [72]. They used a 20 kHz ultrasonic fryer at different power levels (0, 200, 400, 600 and 800 W) to fry the meatballs at 140 and 160 °C for 8, 12 and 16 min. Mechanical properties of the sonicated meatballs had a significant discrepancy from the control group; the hardness and cohesiveness of HIUS-assisted fried samples were substantially lower than the control group which refers to the cavitation phenomenon leading to improved tenderness, while the 800 W samples showed higher hardness comparing to the 200 W treatment showing that higher power of ultrasound can increase the hardness; this may be happened due to faster heating and threading of proteins during cavitation phenomenon leading to development of more firm 3D protein network. For color, after applying ultrasound, an increase in L* value was observed which may be due to caramelization and non-enzymatic reactions.

**Fig. 9:**
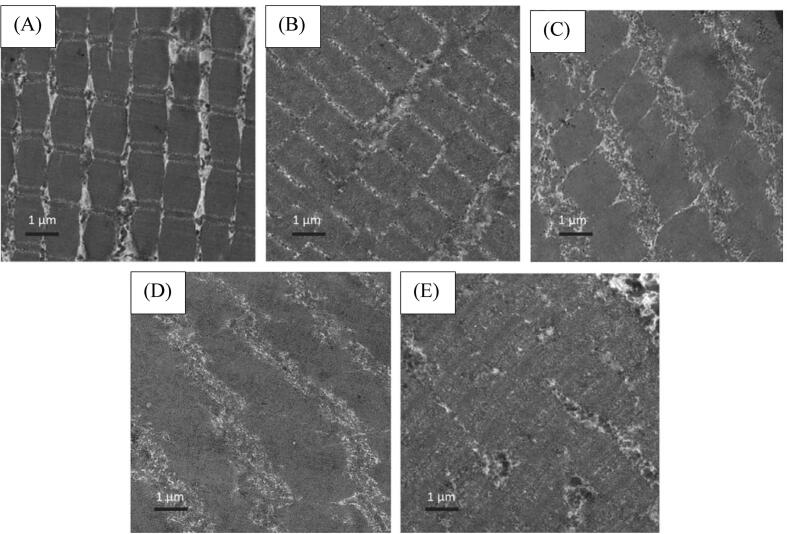
TEM images (3000 ×magnification, bar = 1 μm) of the center of the spiced beef with different ultrasound power with 120 min cooking time: (A) ultrasound power 0 W; (B) ultrasound power 400 W; (C) ultrasound power 600 W; (D) ultrasound power 800 W; (E) ultrasound power 1000 W [71].

The transmission electron microscopy (TEM) was applied to study the microstructure of spiced beef to understand the effect HIUS during the cooking process [71]. Fig. 9 shows the TEM analysis of the center of spiced beef samples treated with different ultrasonic powers when cooked for 120 min. The control presents a more compact myofibre matrix with the relatively complete Z-lines and M−lines (Fig. 9A). While, for the sonicated the samples, the gap between the myofibrils are larger as myofibrils along with the Z-lines were ruptured (Fig. 9B-E). Higher HIUS powers result in more extensive destruction of the myofibrillar framework in beef samples due to the mechanical effects. The cavitation effects of HIUS promote the solubilization of myofibrillar proteins, contributing to the formation of a subtle protein network. Therefore, the M−lines appeared to be weakened and light band was destroyed with the increase in ultrasound power [71]. Some other studies conducted regarding to cooking process by HIUS are presented in Table 3.

**Table 3:** Some studies conducted regarding to promote the meat cooking process by ultrasonication.

Product	Method	Optimal conditions	Results	Reference
Mortadella	Ultrasonic bath	25 kHz, 301 W,00% amplitude, sweep mode of operation, 65 °C to 76 °C	Rapid increase in the internal temperature Enhanced temperature homogeneity No effect on the lipid and protein oxidation speed 30% reduction in cooking time.	[74]
Brown crab (Cancer Pagurus)	Ultrasonic bath with a tank capacity of 55 L.	900 W, 40 kHz, 75 °C, 45 min, heating power of 2000 W	Improved heat transfer Reduction in cooking time up to 15 % Reduced the impact of the meat weight on the heating rate Inhibiting the absorbance of NaCl due to the physical barrier of the crab carapace HIUS couldn’t decrease the microbial loads at low temperature.	[79]
Beef longissimus and pectoralis muscles	Ultrasonic probe in water-filled heating tank.	1000 W, 20 kHz, 70 °C	Improved moisture preservation and texture Decreased flavor intensity as a result of extracting flavor compound in the liquid medium Halved cooking time.	[80]
Frankfurter-type sausages	Ultrasound pretreatment, ultrasonic bath with water	240 W, 25 kHz, 10 sec off/10 sec on cycle, 25 min	50% phosphate reduction leading to increased cooking loss Enhanced textural properties No increase in the lipid oxidation during 21 days storage	[81]

### 4.3 Challenges

However, the studies performed on HIUS-assisted meat and its products cooking confirm that this method can be successfully used, which proves its potential for industrial scale applications, but some challenges should be considered: HIUS-assisted cooking can reduce the cooking loss but higher powers may increase the cooking loss due to the destructive effects of cavitation. Therefore, finding the optimal conditions for HIUS-assisted cooking processes is a necessity; However, the optimal amount of HIUS for a quality factor can be different from the optimal value of HIUS for another quality factor. In this case, the optimal HIUS should be selected with some negligence; There are some concerns in applying the HIUS-assisted cooking technology in an industrial scale, such as the high investment cost, deficiency related to the present regulative agreements, and lack in understanding the consumer satisfactions [82]; The permissible operating temperature of ultrasonic equipment is usually up to 80 °C, this limitation makes it difficult to design the cooking and frying processes of meat products at higher temperatures especially in an industrial scale.

### 4.4 Future trends

HIUS as a non-thermal and green wave, has so advantages and positive impacts in food industry. Nowadays the need of a process in meat industries is obvious. Using HIUS on meat before supplying it to the market can improve the quality of the meat in the next phases. Also, using HIUS on pre-cooked products or in restaurants and caterings can improve their market as well as positive made effects on the properties.

However, more studies are needed to find the optimal conditions to apply in meat products without interfering on their sensorial parameters. To develop these systems on an industrial scale, the impact of HIUS-assisted cooking needs to be evaluated on the attributes which are significant for product quality.

## 5 Ultrasound-Assisted fermentation

Meat and meat products are important sources of protein, fat, essential amino acids, minerals, vitamins and nutrients. Fermented meat products conjointly play a crucial role within the food basket of consumers round the world. From the past to the present, fermentation has been accustomed to modify the chemical, physical, and microorganism compositions of meat products by enzymes and organic catalysts made by microorganisms, as well as yeast and bacteria [83]. In addition to the biological and biochemical changes that fermentation causes in the product, the flavor and texture of the product also change [84]. Fermentation of meat product contains a great impact on the biological value, sensory quality, storage conditions and industrial value of the product, therefore fermentation and its effects are important for each producer and customers [85]. However, fermentation of meat may cause the assembly of poor-quality products and also the presence of undesirable microorganisms and pathogens within the product [86]. In the history of fermentation, microorganisms and bacterium have invariably been used as starter cultures, however with the enlargement of the importance of health and food quality, attention to bacteria in hard meat product has additionally inflated [87].

Recently, the combination of HIUS with fermentation for meat products processing has attracted the attention of researchers. In this regard, *Lactobacillus* species are extensively applied as starter culture for meat fermentation, to improve organoleptic properties as well as bio-preservative tasks [88]. HIUS can be applied in killing infective and undesirable microorganisms, management properties, increasing quality throughout fermentation of meat products and activating the immobilized enzymes which its mechanism of action is increasing the rate of transport of substrates to enzymes [14]. Besides the applications of HIUS in food decontaminations by killing microorganisms using the cavitation phenomenon, this technology has been recently applied to promote the activity of useful microorganisms such as *Lactobacillus* and *Bifidobacterium* using moderate and sub-lethal intensities. This method is used for the production of valuable macromolecules in food [89]. The sub-lethal levels of ultrasonic cavitation may improve fermentative processes by raising mass transfer rates of products and reagents through cell walls due to sonoporation and by activating enzymes and/or modifying cellular metabolism. Fermentation can improve food safety by inhibition/elimination of pathogenic and spoilage-related microorganisms, and produces new flavors and aromas [90], [91]. In addition of the dairy products, probiotic bacteria can be applied in dry fermented meats processed without heat treatment to transfer the probiotics into the human gastrointestinal tract, as well as improving safety and stability, extending shelf life and providing new sensory properties [92]. Probiotics, mainly derived from *Lactobacillus* and Bifidobacterium species, can have beneficial effects on host health. In addition to preventing the growth of spoilage and pathogenic bacteria, *Lactobacillus* promotes organoleptic properties of food and reduces the rate of food spoilage due to the competitive control of spoilage microorganisms.

Almost all the reviewed researches have proved that HIUS at the proper intrinsic and extrinsic conditions is a promising method to promote the fermentation process producing high-quality meat [90], [91], [93], [94]. In comparison with the conventional fermentation, HIUS at sub-lethal powers can stimulate the growth of microorganisms and the activities of enzymes during fermentation which is expected to simultaneously improve the quality factors of the fermented products comprising flavor, taste, texture and color. HIUS has many physical and chemical effects on microorganisms, as well as dilution of cell membranes, surface heating, and breaking down water molecules [95]. The impact of HIUS on microorganisms depends on several factors, the foremost important of that are the type of microorganism, frequency, intensity, duration of sonication and characteristics of the food [96]. So, knowing the type of microorganism used as starter culture is important.

**Fig. 10:**
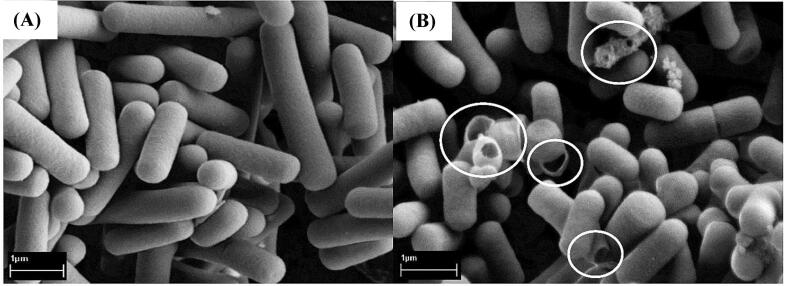
Scanning electron micrographs of: (A) probiotics (*L. casei* ATCC 393, *Lactobacillus* sp. FTDC 2113, *B. longum* FTDC 8643 and *Bifidobacterium* sp*.* FTDC 8943) without the HIUS treatment and (B) the probiotics treated by HIUS (30 kHz, 100 W) adopted by Yeo and Liong [100], circles demonstrate ruptured cells and cells with pores.

*Lactobacillus* bacteria are usually used as a result of their acidity that lowers the pH scale of meat. The foremost vital advantage of this kind of bacteria is to forestall the expansion of pathogenic microorganisms [97]. The pH of soured meat improves the sensory parameters of the product, together with texture, color, and aromatic compounds [98]. Fig. 10 demonstrates the impact of HIUS on *Lactobacillus* bacteria, provided by SEM. Fig. 10(A) suggests the cells that had been now no longer treated, that those cells have a clean and wholesome surface, at the same time as with inside the remedy mattress cells proven in Fig. 10(B), they are torn. Four mechanisms are proposed responsible for fermentation modulation by HIUS [91], [99]:

• Increasing membrane permeability by forming pores and improving nutrient uptake leading to increasing activity of starter culture;
• Increasing enzyme release by improving mass transfer and cell rupture leading to promoting enzyme activity;
• Increasing mass transfer due to cavitation and raising enzymatic reactions leading to increasing interactions between enzyme-substrate;
• Sonochemical and physical modification of food substrates leading to promoting the use of substrate by starter cultures.

**Fig. 11:**
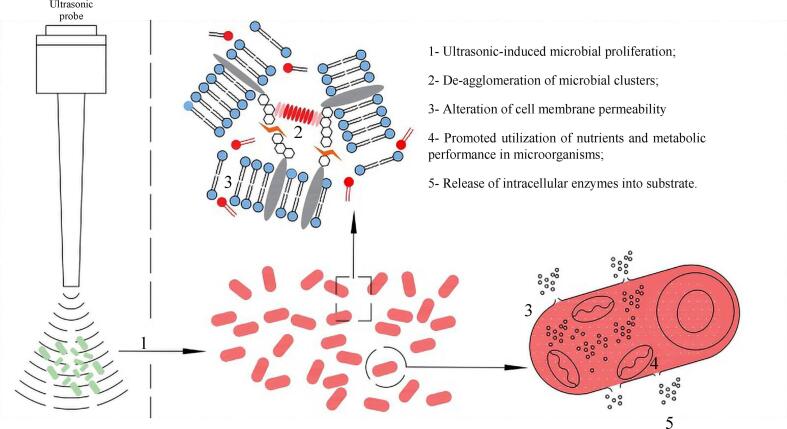
The proposed mechanisms by Yu et al. [94] for HIUS stimulation on microorganisms.

According to Yu et al. [94], the cell membranes alteration is due to the synergistic effects of mechanical vibrations and free radicals including H⋅ and ⋅OH induced by HIUS. The produced free radicals during sonication demonstrate a fundamental role in lipid bilayer relocation and the membrane disruption arising from lipid peroxidation leading to the increase in membrane fluidity and permeabilization consequently accelerating the substances and raising the growth rate of microorganisms [94] (Fig. 11).

When HIUS is applied at low levels of intensity, due to rapid and temporary local pressure changes in the medium arising from the longitudinal ultrasonic waves, the phenomena of diffusion, micromassaging and microflows are induced in meat tissue and cells. At higher levels of intensity, the ultrasonic wave induces radiation force, shock waves, strain, microjets and microstreaming as well as generation of free radicals which deals with the chemical effects of HIUS in aqueous solutions [93].

### 5.1 Application of ultrasound-assisted fermentation in the meat industry

There is little research on application of HIUS in production of fermented meat products. This means that there is a great potential for research into the applications of HIUS for meat and meat products fermentation processes. However, the application of HIUS to raise the growth rate of microorganisms is considered undervalued [101].

The composition of the meat undergoes a different fermentation process, as it contains important amino acids and peptides but has small amounts of fermentable sugars. Lactic acid bacteria (LAB) make the specific attributes of these products, by acidification and the production of organic acids, improving taste, flavor and biological safety [91]. HIUS can induce an increase in the membrane permeability and optimal growth of *Lactobacillus*, which is used as strain to produce lactic acid, leading to the reproduction of microbial cells which results in the production of secondary metabolites. It is worth mentioning that in fermented meat products, lower power HIUS in the ranges of sub-lethal levels must be applied which requires a strict control of the operation conditions during the fermentation process.

Ojha et al. [89] used the HIUS technology in a meat model system to study the behavior of *Lactobacillus* in the fermentation process. They prepared meat extract by cooking minced beef in water and then centrifugated the obtained mixture. MRS broth and meat extract solution *Lactobacillus sakei* (1×10^6^ cfu/mL) culture was sonicated using a 20 kHz, 550 W ultrasonic probe with a diameter of 13 mm. They found that the HIUS treatment could control the growth rate of *L. sakei* as evident from growth model parameters obtained by fitting the Gompertz model. They finally proposed HIUS can be applied for stimulation and/or retardation of *L. sakei* growth depending on the sonication conditions which can be extended to other probiotics.

In the process of dry fermented sausage production and in the fermentation stage, a series of physical, biochemical and microbiological modifications occur leading to the improvement of the characteristic appearance, flavor, and aroma of the product, as well as preservation and food safety [84], [102]. In the production process, proteases, lipases and bacterial origin are essential for the development of flavor [103]. HIUS has sufficient capability to promote the activity of proteases and lipases which subsequently leads to the improvement of releasing fatty acids, amino acids and formation of volatile and aromatic compounds [102]. They applied the HIUS treatment, immediately after the meat batter was embedded and studied its effects on the free amino acid profile and volatile compounds of the fermented sausage during its shelf life. The meat batter samples were sonicated for 0, 3, 6, and 9 min, with the power of 128 W and frequency of 25 kHz. They reported the sonicated sausages showed higher isoleucine and phenylalanine values compared to the control. The sonicated sausages showed higher free amino acids values compared to the non-sonicated samples at the end of manufacture (day 28 of ripening/day 1 of storage) and storage (day 120). This is due to the cavitation which damaged the structure of cellular organelles leading to the release of calcium from the sarcoplasmic reticulum and cathepsin enzymes from lysosomes and subsequently activating the calpain system. In addition, raising temperature of the medium due the sonication process led to the increased activity of proteases. To provide the desirable effect of HIUS on the fermentation process of sausage, the HIUS treatment conditions should be optimized, including intensity, sonication time, and temperature as well as the medium characteristic including viscosity, fat content and water content, etc. The cavitation bubbles produce reactive oxygen species leading to the increase in lipid oxidation and the formation of ketones, alcohols and aldehydes in the fermented sausage. This is due to the formation of free radicals during sonication, which can easily react with oxidizable compounds [102], [104]. de Lima Alves et al. [102] reported the HIUS treatment led to the higher values of pentanal, hexanal, ethanol and hexanol in the fermented sausages than to the control samples. Therefore, applying HIUS during meat batter preparation of dry fermented sausages can significantly affect the fermentation process and the formation of volatile compounds derived from lipid oxidation. However, it is worth mentioning that these changes should lead to the improved sensory properties and consumer satisfaction.

L. de Lima Alves [101] investigated the effect of HIUS exposure times on the fermentation of Italian salami. The growth of starter bacteria, LAB and *Micrococcaceae* during processing and storage of the Italian salami samples were studied. They used a 25 kHz ultrasonic bath with nominal power 500 W and sonicated the samples for 3, 6 and 9 min. They reported HIUS improved the growth of LAB compared to the control. They proposed several mechanisms of action of HIUS on microbial growth: the ability of HIUS to deagglomerate microorganisms and promote their viability; changes in permeability and selectivity of the cell membrane due to the formation of a temporary pore leading to the release and transport of oxygen and nutrients; agitation due to the cavitation leading to the improved substances transport; breakage resulting in increased bioavailability of nutrient macromolecules and self-defense mechanism against the biotic and abiotic mechanical stress caused by HIUS. The HIUS treatment with 9 min sonication time demonstrated higher bacteria counts after 120 days of storage, this delayed effect was due to the cavitation breakage of macromolecules resulting in increase in the nutrient availability [105] that may be absorbed only after a long period. Although, it can be confidently concluded that HIUS impacts on the growth of *Micrococcaceae* and LAB, interactions of other factors such as initial microbial salt content, oxygen availability and water activity should not be neglected. HIUS has led to the increase in pH after 30 days and 120 days storage of Italian salami [101]. This is due to releasing proteolytic enzymes and increasing the amino acids availability.

Stadnik et al. [92] studied the effect of sonication and inoculation with *Lactobacillus casei* ŁOCK 0900 on the proteolysis process of dry-aged pork loins. The effects of HIUS on total viable counts (TVC) and the number of LAB in dry-aged meat cuts were studied. After salting, the trimmed loins (*M. longissimus thoracis*) from commercial cross-bred pigs were sonicated in a 40 kHz ultrasound bath with intensity of 2.5 W.cm^−2^ for 120 s. They sprayed one of the sonicated batches of loins with the inoculum of *L. casei* ŁOCK 0900 to reach the initial level of 10^6^– 10^7^ CFU. g^−1^ meat and stored in a laboratory ageing chamber with a relative humidity of 75%-80% and temperature of 16 °C for 28 days. The HIUS processing of raw meat did not affect the total viable counts (TVC) and the L* color value, but the a* color value sonicated samples were slightly higher than the control. The HIUS treatment significantly influenced the proteolytic breakdown of muscle proteins and nonprotein nitrogen content confirming the usefulness of HIUS in acceleration of the ageing process [106]. The sonicated loins inoculated with *L. casei* ŁOCK 0900 demonstrated the value of proteolysis index and significantly different pH and water activity. They concluded the HIUS treatment followed by inoculation with a probiotic strain *L. casei* ŁOCK 0900 is an effective method to speed up the proteolytic changes in dry-aged loin cuts.

Wójciak et al. [93] studied the effect of HIUS treatment on the oxidative stability of uncured organic fermented beef. They focused on thiobarbituric acid reactive substances (TBARS) and fatty acid (FA) profile of the fermented beef during ripening. They immersed the beef samples in the cold acid whey (4–6 °C) in a 40 kHz ultrasonic batch processor with acoustic power of 480 W and sonicated the beef samples for 10 min. The final temperature of the acid whey reached to 19 ± 1 °C. The fermented beefs were tested during the ripening process at 31, 62, and 93 days. TBARS are the major oxidation products of polyunsaturated fatty acid during storage of fermented beef. They found that HIUS treatment affected the TBARS factor after 31 and 62 days of ripening which is due to rising the formation rate of free radicals easily reacting with lipids and proteins [104].The sonicated fermented beefs had a greater quantity of fatty acid, while showed the lowest participation of the red and yellow color in the general tone of the color at the end of storage time.

Ojha et al. [88] investigated the effect of HIUS frequency and *L. sakei* culture on the nutritional and physicochemical attributes of beef jerky. The cultured samples were exposed to the HIUS treatment at frequencies 25, 33 and 45 kHz for 30 min in ultrasonic bath systems at a temperature of 30 °C, were cured for 18 h at 4 °C and then were dried using a hot air dryer at 60 °C for 4 h. They observed no significant difference between true protein content of control cultured and uncultured samples but observed significant differences for HIUS-treated samples. The synergic effects of *L. sakei* and HIUS resulted in hydrolysed beef proteins and short chain polypeptides or free amino acids. The highest value of amino acid content belonged to the cultured beefs treated with 25 kHz HIUS, which confirms the effectiveness of HIUS on protein hydrolysis. This is due to the breakdown of beef proteins occurred due to the culture and HIUS treatments. While, the frequency of 25 kHz yielded the highest values for cysteine, 33 kHz for proline and glycine and 45 kHz for alanine, glutamic acid, phenylalanine and valine. These results confirm that the frequency of HIUS plays a key role in the meat fermentation process, while there are limitations in maneuvering on the ultrasonic frequency. It has been proven that HIUS can increase taurine content in beef jerky due to the releasing bound taurine arising from sonochemical mechanisms. Taurine is a sulphur-containing *β* amino acid having various biological and health benefits and protective roles [88]. Table 4 presents some applications of HIUS-assisted fermentation in the meat industry.

**Table 4:** Some applications of HIUS in fermentation of meat and meat products.

Product	Method	Optimal conditions	Results	Reference
Yak meat	Ultrasound pretreatment using a probe-based ultrasonic processor; 200, 300 and 400 W	20 kHz, 300 W, 30 min, 25 °C	Decrease in moisture content and hardness Shear force significantly decreased with the increase of ultrasonic power HIUS negatively affected the meat’s color, smell, and taste. Increase in tenderness, alcohols and aldehydes contents the appropriate ultrasonic power of 300 W particularly improves quality of dry-cured yak meat.	[107]
Uncured dry fermented beef	Ultrasound cold bath; immersion in in acid whey environment (4 °C), 5 and 10 min sonication time	40 kHz, 480 kW, 10 min	The effect of sonication on biogenic amine (BA) formation was investigated positive effect on retarding histamine (HIS), cadaverine (CAD), tyramine (TYR) and putrescine (PUT) formation the sonication treatment did not inhibit the growth of lactic acid bacteria (LAB) in dry-fermented beef	[108]
Beef jerky	Ultrasonic bath systems, *Lactobacillus* sakei culture, 30 °C; 25, 33 and 45 kHz, 30 min sonication time	They did not propose optimal conditions, Changes in fatty acid profiles were significantly affected by individual and/or interactive effects of L. sakei, drying time and ultrasonic frequency	Changes in fatty acid profiles were significantly affected by individual effected of *L. sakei*, drying time and ultrasonic frequency. Interaction effect of ultrasound frequency with culture and drying time were more pronounced compared to individual effects.	[109]

### 5.2 Challenges

HIUS demonstrates a great capability to be an alternative or assistant to the conventional heat-based technologies that have a high ability to be utilized in various food industry processes as well as fermentation. However, despite all the benefits of using ultrasound in fermentation, there are challenges during this direction, including: Intrinsic factors including type of microorganism and medium and composition and extrinsic factors including HIUS frequency and power, treatment time and temperature indicate the degree of HIUS effectiveness on microorganisms, which make the studies to achieve different and somewhat contradictory results. This issue highlights the need of protocol and standard for the use of HIUS in meat fermentation; The utilization of HIUS causes an increase in temperature during the process which may have a detrimental effect on the organoleptic properties of the meat product; HIUS has not been completely successful in accelerating the activities of microorganisms and enzymes, but because of the application of temperature and pressure during fermentation by ultrasound, acceptable results are obtained; If the pressure and temperature in the fermentation process are not optimal, free radicals are created in the product in conjunction with HIUS leading to the damage to the protein structure of the product; It should be noted that HIUS accelerates lipid oxidation, therefore applying this technology in the preparation of meat bather for product manufacturing should be carefully considered to avoid undesirable effects on the flavor and sensorial properties of the product.

### 5.3 Future trends

Despite recent advances in HIUS-assisted meat fermentation, higher knowledge of molecular, microscopic, and physicochemical changes during the process is required to design equipment and procedures that combine HIUS with conventional technologies, to improve the quality of meat products and overcome the limitations of HIUS application in commercial scale fermentation. More research should be conducted to study the influence of HIUS on the wide range of natural meat microflora to assess the applicability of this technology to promote the hygienic status of meat during fermentation. Therefore, a lot of research should be conducted aiming to optimize the influential factors affecting the HIUS- assisted fermentation process of meat and meat products. Eventually, there is lack of research on applying the HIUS technology in the meat fermentation processes and hence further studies should be conducted to provide a reliable commercialization of this technology for use in the food industry.

## 6 Conclusion

Nowadays there is a growing interest in non-thermal food processing methods leaving least effects on the nutritional and sensory properties of foods. Since the freezing, cooking and fermenting processes meat products are time consuming and require a lot of energy, HIUS has come to the attention of scientists as an assistant non-thermal technology and it has been proved that this technology has a significant role in accelerating and promoting these processes which leads to the production of better-quality products than the traditional methods. The mechanical effects produced by HIUS are important phenomena that are responsible for promoting the freezing/thawing, cooking and fermentation processes and improving the quality of the final products. Nevertheless, there are still many challenges to develop HIUS-based equipment for fresh meat processing. The effect of HIUS on meat processing has rarely been associated with adverse effects on meat. Nevertheless, some mild adverse effects of this technology on various properties of the meat under processing have been reported, which are needed further studies to be confirmed. On the other hand, the complex matrix of meat makes HIUS transmission difficult. Differences in the in the ultrasonic parameters and sample properties (including muscle composition, type of muscle, dimensional characteristics, etc.) make it difficult to recommend a most suitable conditions for quality improvement in such processes. therefore, standardization of the HIUS-assisted meat processing and presentation of corresponding regulative agreements are highly recommended. Further research may be necessary to study the effects of HIUS on meat processing, and its combination with other technologies. Also, HIUS needs to be scaled up for industrial applications. Finally, the use of HIUS as an effective method to ensure the safety of meat processing and improve the processes of this industry is recommended to manufacturers.

## Declaration of Competing Interest

The authors declare that they have no known competing financial interests or personal relationships that could have appeared to influence the work reported in this paper.
